# Draft Genome Sequences of *Leptospira santarosai* Strains U160, U164, and U233, Isolated from Asymptomatic Cattle

**DOI:** 10.1128/genomeA.00910-15

**Published:** 2015-08-13

**Authors:** Frederico S. Kremer, Marcus R. Eslabão, Monize Provisor, Rafael D. S. Woloski, Osmar V. Ramires, Luisa Z. Moreno, Andrea M. Moreno, Camila Hamond, Walter Lilenbaum, Odir A. Dellagostin

**Affiliations:** a Centro de Desenvolvimento Tecnológico, Núcleo de Biotecnologia, Universidade Federal de Pelotas, Pelotas, Brazil; b Universidade de São Paulo, São Paulo, Brazil; c Universidade Federal Fluminense, Rio de Janeiro, Brazil

## Abstract

In the present work, we announce the draft genomes for three new strains (U160, U164, and U233) of *Leptospira santarosai*, isolated from urine samples from asymptomatic cattle in Rio de Janeiro, Brazil.

## GENOME ANNOUNCEMENT

The *Leptospira* genus comprises at least 22 species, of which 15 are classified as pathogenic, including *L. interrogans*, *L. borgpetersenii*, and *L. santarosai* (1–3). Leptospirosis is a reemerging worldwide-distributed zoonosis and may occur as asymptomatic or in an acute form (4–6). There is a lack of information about the host-pathogen interactions during leptospirosis (7). Therefore, the availability of new genomic sequences, covering different species, hosts, and manifestations of the disease, may provide very useful data to carry out comparative genomics and for better understanding the pathogen (8).

In the present work, we performed whole-genome sequencing of the strains U160 (serogroup Sarmin), U164 (serogroup Tarassovi), and U233 (serogroup Grippotyphosa) of *Leptospira santarosai*, isolated from urine samples of asymptomatic cattle in Rio de Janeiro, Brazil, previously described by Hamond et al. (9). The whole-genome shotgun sequencing was performed using the Illumina MiSeq platform with a paired-end library at Macrogen. All three genomes were *de novo* assembled using A5 (10), SGA (11), Ray (12), and CISA (13). For the genome annotation, open reading frames (ORFs) found by Prodigal (14) were aligned against a data set of proteins from the *Leptospira* genus from Uniprot (http://www.uniprot.org) using BLAST (15, 16) and against the AntiFam database (17) using HMMER (18). The tRNAs and rRNAs were predicted using tRNAscan-SE (19) and RNAmmer (20), respectively. For the prediction of other noncoding RNAs (ncRNAs) and regulatory sequences, a combination of BLASTn and Infernal (21) searches using the Rfam database (22) was used. A multilocus sequence typing (MLST) analysis was performed using BLASTn searches against the *Leptospira* MLST repository (http://leptospira.mlst.net).

The assembly resulted in 204 scaffolds (length, 4.2 Mb; *N*_50_, 40,346) for U160, 169 scaffolds (length, 4.1 Mb; *N*_50_; 72,508) for U164, and 152 scaffolds (length, 4.0 Mb; *N*_50_, 79,752) for U233. Using our annotation pipeline it was possible to identify 3,955 coding sequences (CDSs), 38 tRNAs, 4 rRNAs, 1 ncRNA, and 1 riboswitch on U160, 3,799 CDSs, 37 tRNAs, 4 rRNAs, 2 ncRNAs, and 2 riboswitches on U164, and 3,634 CDSs, 37 tRNAs, 6 rRNAs, 6 ncRNAs, and 2 riboswitches on U233. In the MLST analysis, U160 showed unique alleles for all the 7 loci, while U233 showed for only one locus and U164 had a perfect match for all loci. All isolates have unique allelic patterns if compared to the reference database, although a nearest-match search identified both U164 and U233 as close to the *L. santarosai* strain Aa 3. The uniqueness observed in these isolates might provide new information about the molecular variability and distribution of *L. santarosai* in South America.

### Nucleotide sequence accession numbers.

These whole-genome shotgun projects have been deposited at DDBJ/EMBL/GenBank under the accession numbers LAYP00000000 for U160, LAZM00000000 for U164, and LAZN00000000 for U233. The versions described in this paper are LAYP01000000, LAZM01000000, and LAZN01000000, respectively.
